# Supplementary material for: Enteric nervous system degeneration in human and murine CLN3 disease, is ameliorated by gene therapy in mice
Source: Acta Neuropathol Commun. 2025 Dec 22;13:260. doi: 10.1186/s40478-025-02205-7 (PMC12751165; doi:10.1186/s40478-025-02205-7)
Supplement: Supplementary file 3 — Supplementary Material 1 [file 40478_2025_2205_MOESM3_ESM.docx]

**Figure S1**


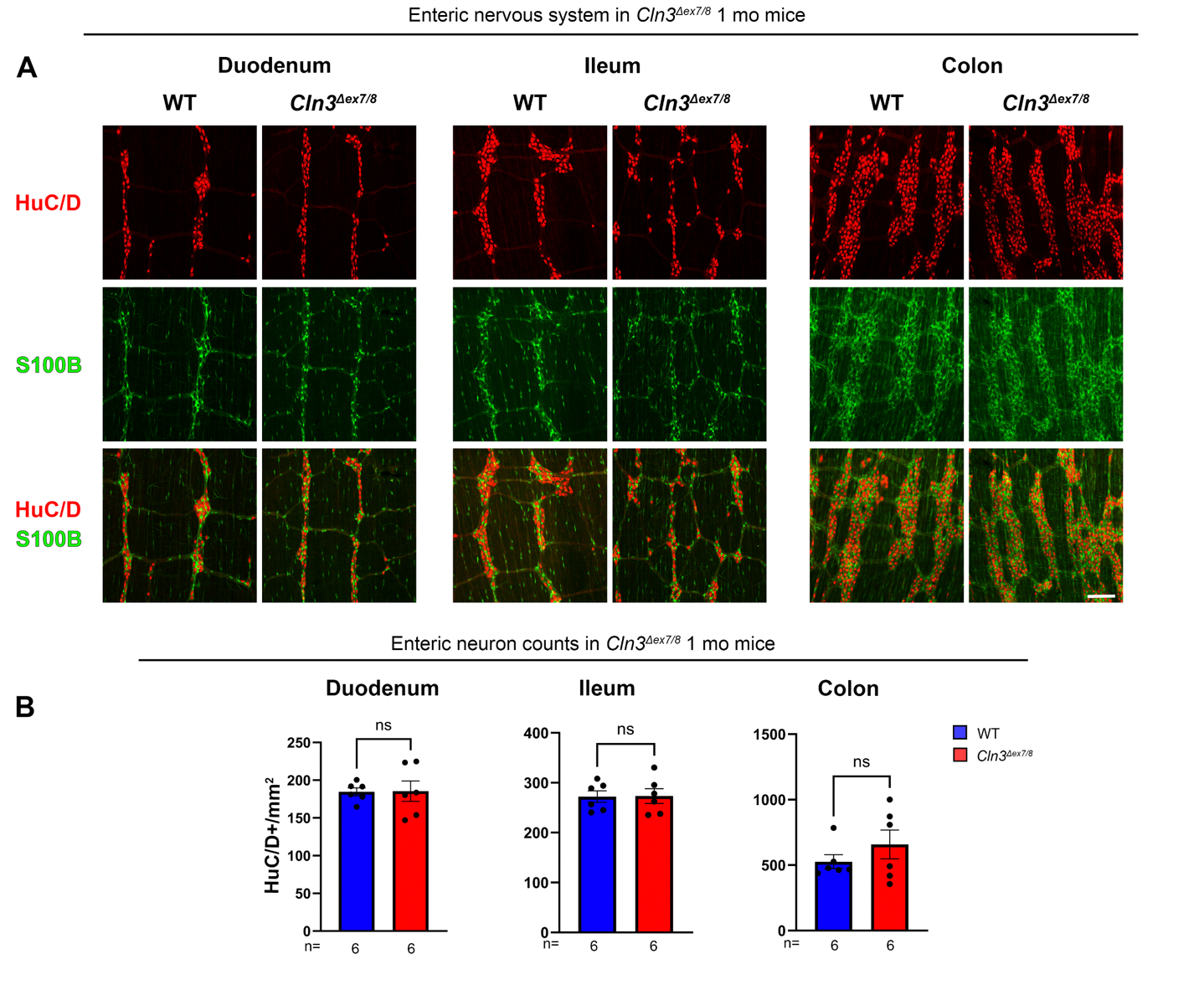


**Figure S1. Lack of readily detectable enteric nervous system pathology at 1 month of age. (A)** Immunostaining for HuC/D (neurons, red) and the glial marker S100B (green) reveal the myenteric plexus of 1 month old *Cln3^Δex7/8^* mice appears similar to age matched WT mice in duodenum, ileum and colon. Scale bar 200µm. **(B)** Counts of the density of HuC/D+ neurons reveal no significant difference in any bowel regions of 1 month old *Cln3^Δex7/8^* mice compared to age matched WT controls. Unpaired t-tests **(B)**. Data ± SEM.

**Figure S2**


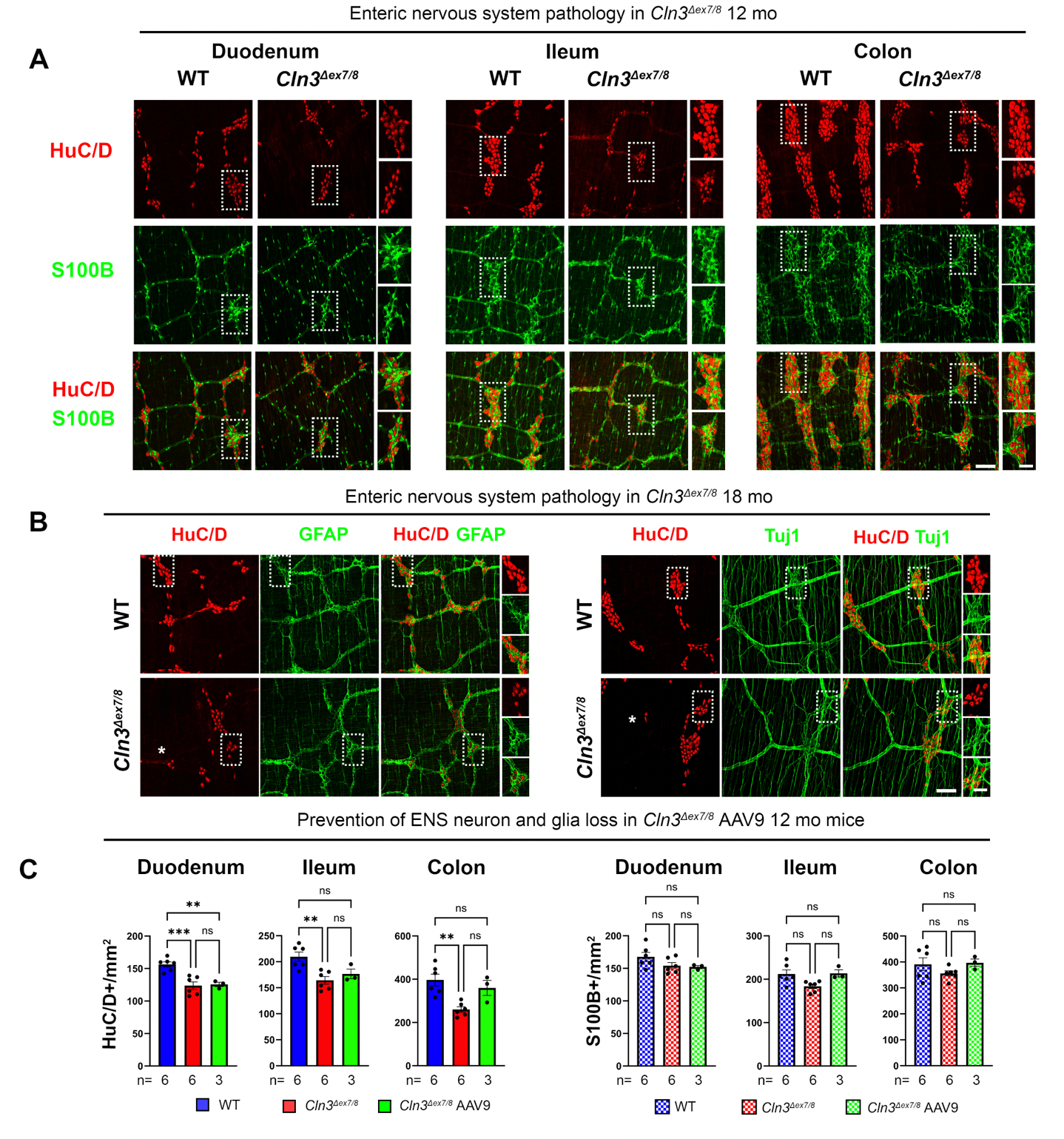


**Figure S2. Evidence for enteric nervous system pathology in *Cln3^Δex7/8^* mice at 12 and 18 months. (A)** Photomicrographs show immunostaining for HuC/D (neurons, red) and the glial marker S100B (green) in the duodenum, ileum and colon *Cln3^Δex7/8^* mice and age-matched wildtype (WT) controls at 12 months of age (mo). Scale bars 200µm, 50µm in higher magnification views. **(B)** Photomicrographs showing immunostaining for HuC/D (neurons, red) and either the glial marker glial fibrillary acidic protein (GFAP, green) or the neuronal microtubule marker Tuj1 (green) in the duodenum *Cln3^Δex7/8^* mice and age-matched WT controls at 18 months of age. Scale bars 200µm, 50µm in higher magnification views. Asterix (*) indicates regions of near complete enteric neuron loss. **(C)** Measurements of the density of HuC/D+ myenteric neurons and S100B+ myenteric glia reveal no significant differences (ns) in all bowel regions of 12-month-old *Cln3^Δex7/8^* mice treated with AAV9.hCLN3 vs. untreated *Cln3^Δex7/8^* mice. Data ± SEM. One-way ANOVA with a post-hoc Bonferroni correction, ** p≤0.01, *** p≤0.001.
